# Two Subunits of the Rpd3 Histone Deacetylase Complex of *Cochliobolus heterostrophus* Are Essential for Nitrosative Stress Response and Virulence, and Interact With Stress‐Response Regulators ChHog1 and ChCrz1

**DOI:** 10.1111/mpp.70131

**Published:** 2025-08-07

**Authors:** Jinyu Fan, Jun Hu, Dan Li, Yuanyuan Tian, Mengjiao Jia, Tianye Liang, Hongyu Pan, Xianghui Zhang

**Affiliations:** ^1^ College of Plant Science Jilin University Changchun Jilin China

**Keywords:** ChPho23, ChSds3, *Cochliobolus heterostrophus*, nitrosative stress, virulence

## Abstract

Southern corn leaf blight (SCLB), caused by *Cochliobolus heterostrophus*, is a destructive disease in maize‐growing areas worldwide. Reactive nitrogen species derived from nitric oxide exhibit antimicrobial activities by interacting with microbial cellular components, leading to nitrosative stress in pathogens. However, the regulatory mechanisms underlying adaptation to nitrosative stress remain largely unexplored in *C. heterostrophus*. In this study, two components of the Rpd3 histone deacetylase complex, ChPho23 and ChSds3, were identified as being involved in the nitrosative stress response and virulence in *C. heterostrophus*. ChPho23 and ChSds3 are not only required for vegetative growth and conidiation but are also essential for responding to oxidative stress. ChPho23 and ChSds3 directly interact with ChHog1, and ChHog1 in turn interacts with ChCrz1 to up‐regulate the transcription of genes involved in the nitrosative stress response, which enable *C. heterostrophus* to cope with nitrosative stress. Furthermore, mutants of Δ*Chhog1* and Δ*Chcrz1* exhibited significantly reduced virulence on detached maize leaves and increased sensitivity to nitrosative stress. Taken together, these findings indicated that ChPho23 and ChSds3 are crucial for fungal growth, conidiation, nitrosative stress response, and virulence in *C. heterostrophus*. This knowledge could be applied to the design of strategies that target ChPho23 and ChSds3 for controlling SCLB.

## Graphical abstract

ChPho23 and ChSds3, two components of the Rpd3 histone deacetylase complex associated with ChHog1, regulate the transcription factor ChCrz1 and modulate the nitrosative stress response and virulence in *Cochliobolus heterostrophus*.

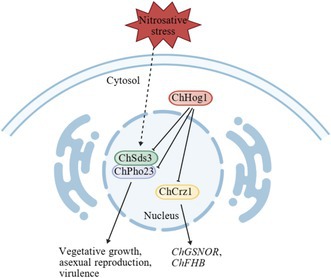

## 1 Introduction

Nitric oxide (NO) is a highly diffusible signalling molecule in several biological systems, playing a crucial role in immune responses of both plants and animals (Bogdan 2015; Jian et al. 2021; Lundberg et al. 2015; Yun et al. 2016). Reactive nitrogen species derived from NO exhibit antimicrobial activities by interacting with microbial cellular components, leading to nitrosative stress (NS) in pathogens (Jian et al. 2021). The biochemical properties of NO, such as its membrane permeability and ability to target intracellular protein thiols and metal centres, make it an effective antimicrobial molecule with broad activity against diverse pathogens. Induced NO levels produced by host can significantly impact microbial physiology and metabolism (Urbano et al. 2018). In contrast, pathogens employ various strategies to counteract NS during the infection process (Arasimowicz‐Jelonek and Floryszak‐Wieczorek 2014; Schlicht and Kombrink 2013). In *Fusarium graminearum*, transcription factor FgAreB recruits the chromatin‐remodelling complex SWI/SNF to the promoters of genes involved in the NS response to modulate fungal NS response (Jian et al. 2021). Additionally, some regulators have been characterised in pathogenic bacteria and human‐pathogenic fungus *Candida albicans* (Arasimowicz‐Jelonek and Floryszak‐Wieczorek 2014; Chiranand et al. 2008; Tucker et al. 2010; Urbano et al. 2018). However, the regulatory mechanisms remain largely unexplored in phytopathogenic fungi, particularly in Dothideomycetes, which encompasses a large group of plant pathogens.

Histone acetylation and deacetylation are among the most extensively studied epigenetic modifications, catalysed by histone acetyltransferases (HATs) and histone deacetylases (HDACs). Moreover, HATs and HDACs play crucial roles in development and virulence in pathogenic fungi (Bauer et al. 2016; Fuks et al. 2000; Gu et al. 2022; Jeon et al. 2014; Kong et al. 2018; Lopes da Rosa et al. 2013; Soukup et al. 2012; Xin et al. 2013). Rpd3 is a class I HDAC that acts as a key component of the Rpd3L or Rpd3S complex, which consists of multiple subunits in *Saccharomyces cerevisiae* (Kurdistani and Grunstein 2003; Yang and Seto 2008). In *Fusarium pseudograminearum*, the Rpd3L‐like HDAC complex contains at least eight subunits, including FpRpd3, FpSds3, FpPho23, FpSin3, FpRxt3, FpCti6 and FpUme6 (Zhang et al. 2020). To date, some subunits of the Rpd3 complex have been studied in filamentous fungi. MoRpd3 regulates reproduction and pathogenic development in the rice blast fungus *Magnaporthe oryzae* (Lee et al. 2021). The Δ*Rpd3* shows reduced growth, conidiation, and virulence in *Alternaria alternata* (Ma et al. 2021). Sin3, another component of the Rpd3 complex, acts as a transcriptional repressor of *ATGs* and a negative regulator of autophagy induction in the rice fungal pathogen *M. oryzae* (Wu et al. 2023). Furthermore, the Sin3 histone deacetylase complex is required to maintain H3K27me3 occupancy and stable gene repression by directly interacting with P55, a subunit of polycomb repressive complex 2 (Lin et al. 2022). In the *C. albicans* , the Rpd3 HDAC complex component Pho23 is involved in the transcriptional regulation of autophagy, cell wall stress response and pathogenicity (Du et al. 2023). The Rpd3 HDAC complex, in association with the ING protein Fng2, regulates fungal development and pathogenesis in *Fusarium graminearum* (Xia, Wang, et al. 2024). However, the roles of the Rpd3 HDAC complex in *Cochliobolus heterostrophus*, the causal agent of southern corn leaf blight, remain largely unknown.

The high osmolarity glycerol (HOG) mitogen‐activated protein kinase (MAPK) pathway is highly conserved in fungi and plays an important role in responding to environmental stresses, particularly osmotic signals (de Nadal and Posas 2022; Roman et al. 2020; Wang et al. 2023; Xia, Xia, et al. 2024). In most cases, the HOG pathway functions as an intermediate that transmits signals to downstream transcription factors. Li et al. (2023) demonstrated that the basic leucine zipper transcription factor Atf1 is a downstream component of Hog1, responsible for blue light responses, conidial germination, vegetative growth and oxidative stress resistance in *Trichoderma guizhouense*. Additionally, the calcium‐calcineurin and HOG signalling pathways exhibit a synergistic effect in regulating the pathogenicity of *F. graminearum* and its sensitivity to tebuconazole and other stresses (Wang et al. 2023). In *Fusarium verticillioides*, FvHog1 directly phosphorylates the Ca^2+^‐responsive transcription factor (FvCrz1) to regulate Ca^2+^ homeostasis (Xia, Xia, et al. 2024). The transcription factor Calcineurin‐Response Zinc finger 1 (CRZ1) directly regulates approximately 300 genes in various stress pathways, making it a key player in host–pathogen interactions (Cacciotti et al. 2024). As previously elucidated, Crz1 is involved in fungal growth, development, and pathogenicity (Cacciotti et al. 2024). The absence of Crz1 causes obvious defects in the vegetative growth and virulence of many phytopathogenic fungi. A prime example is the Δ*Fgcrz1* mutant of *F. graminearum*, which exhibits slower hyphal growth and reduced virulence on flowering wheat heads and maize silks (Chen et al. 2019). Furthermore, Crz1 controls growth, development and full virulence in *M. oryzae* and *Botrytis cinerea* (Schumacher et al. 2008; Zhang et al. 2009). Microsclerotia and fruiting body formation, both crucial to fungal life, are intimately linked to Crz1. For instance, the Δ*Vdcrz1* mutant fails to generate microsclerotia (Xiong et al. 2015), while the Δ*Vpcrz1* mutant displays impaired fruiting body formation (He et al. 2016). In *F. verticillioides*, a noteworthy observation is that FvHog1 can directly phosphorylate FvCrz1, promoting the transport of phosphorylated FvCrz1 into the nucleus to regulate Ca^2+^ homeostasis (Xia, Xia, et al. 2024). While most studies on Crz1 have focused on its roles in development and virulence, its function in the NS response has been rarely reported.

In this study, we revealed that nitric oxide (NO) exerts a dramatic inhibitory effect on the growth of *C. heterostrophus*. Here, we demonstrated that ChPho23 and ChSds3, two components of the Rpd3 HDAC complex, interact with ChHog1 to overcome NS. Furthermore, under NS conditions, ChHog1 interacts with transcription factor ChCrz1, which in turn modulates the transcription of *ChGSNOR* and *ChFHB* to respond to NS. These findings provide new evidence and insights into the relationship between the Rpd3 HDAC complex and the Hog1‐MAPK pathway, and highlight the critical role of transcription factor ChCrz1 in regulating response to NS in *C. heterostrophus*.

## 2 Results

### 2.1 Identification of Rpd3 Deacetylation Complex Genes in *C. heterostrophus*

**FIGURE 1:**
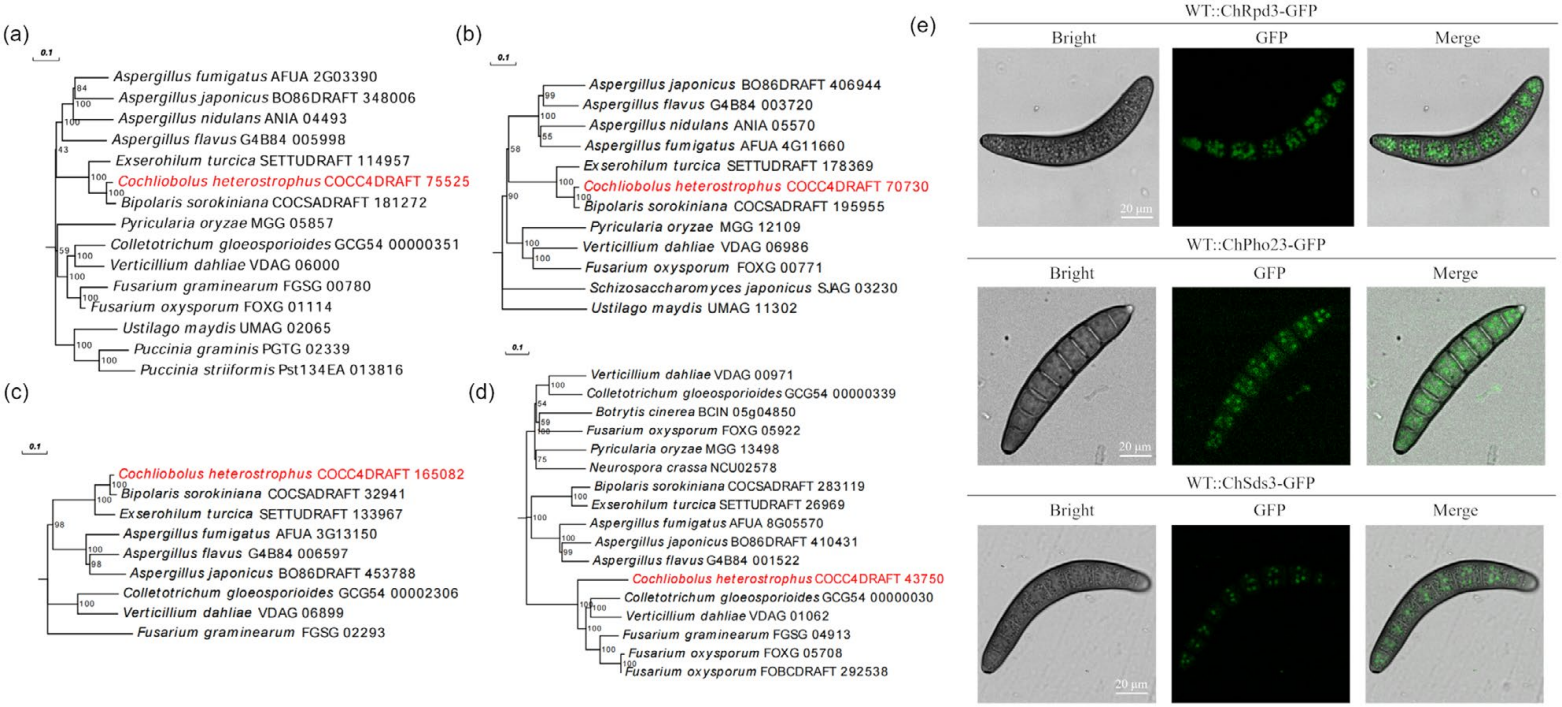
Phylogenetic analysis of ChRpd3, ChPho23, ChSds3 and ChSin3 and subcellular localisation of ChRpd3, ChPho23 and ChSds3. (a) Phylogenetic tree of ChRpd3. (b) Phylogenetic tree of ChPho23. (c) Phylogenetic tree of ChSds3. (d) Phylogenetic tree of ChSin3. The information of ChRpd3, ChPho23, ChSds3 and ChSin3 were analysed by Uniprot (https://www.uniprot.org/), figure was drawn by GPS v. 2.0. (e) The subcellular localisation of ChRpd3, ChPho23 and ChSds3 in conidia. Bar = 20 μm.

To identify the Rpd3 deacetylation complex genes in *C. heterostrophus*, amino acid sequences of Rpd3, Pho23, Sds3 and Sin3 from *Saccharomyces cerevisiae* were used as queries in a BLAST search against the genome of *C. heterostrophus* strain C4. The genes *ChRPD3* (COCC4DRAFT_75525), *ChPHO23* (COCC4DRAFT_70730), *ChSDS3* (COCC4DRAFT_165082) and *ChSIN3* (COCC4DRAFT_43750) were identified. The predicted protein products of ChRpd3, ChPho23, ChSds3 and ChSin3 are 645, 694, 555 and 2508 amino acids in length, respectively. Phylogenetic analysis revealed that the components of the Rpd3 complex are conserved across filamentous fungi (Figure 1a–d). To investigate the subcellular localisation of Rpd3, Pho23 and Sds3, Rpd3‐GFP, Pho23‐GFP and Sds3‐GFP fusion constructs were introduced into *C. heterostrophus* wild type (WT). We found ChRpd3, ChPho23 and ChSds3 were located in the nucleus (Figure 1e).

### 2.2 *ChRPD3* , *ChPHO23* , *ChSDS3* and *ChSIN3* Are Required for Vegetative Growth, Asexual Reproduction and Virulence in *C. heterostrophus*

**FIGURE 2:**
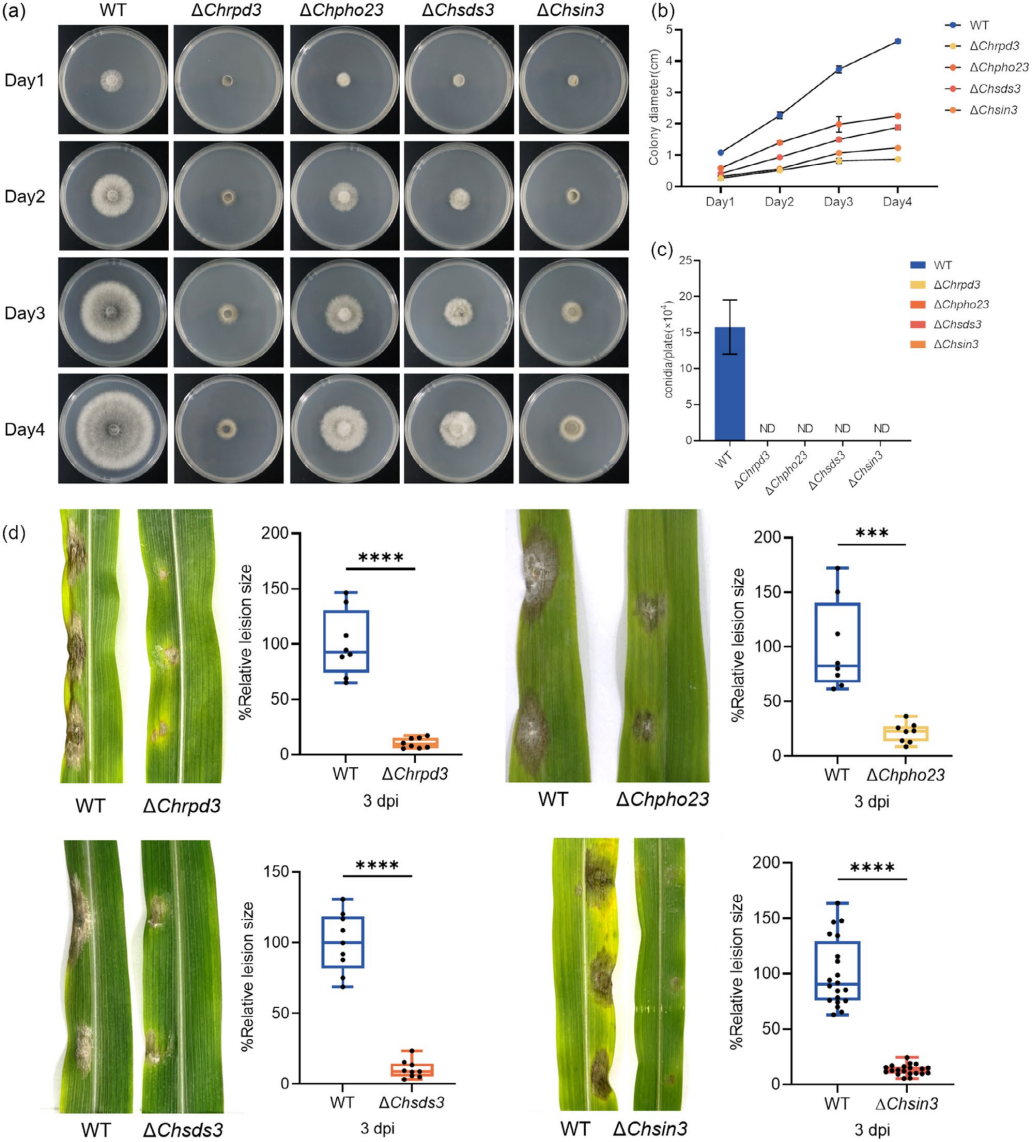
ChRPD3, ChPHO23, ChSDS3 and ChSIN3 are important for vegetative growth, asexual reproduction and virulence in *Cochliobolus heterostrophus*. (a) Colony phenotype of wild‐type (WT) and mutant strains on complete medium with xylose (CMX). (b) Statistical analysis of colony diameter of each strain. (c) Statistical analysis of conidia production of WT and mutant strains. (d) Virulence of WT and mutant strains on maize. Inoculation with a 5‐mm mycelial plug of indicated strains on detached susceptible *Zea mays* ‘B73’ leaves. Statistical analysis of the lesion size of the indicated strains on detached maize leaves. Error bar represented standard deviation (SD) from mean at least three independent experiments (marked with black dots on the bars). *** in (d) indicates significant differences based on *t* test (*p* < 0.001). **** in (d) indicates significant differences based on *t* test (*p* < 0.0001).

To determine the roles of *ChRPD3*, *ChPHO23*, *ChSDS3* and *ChSIN3* in mycelial growth, mycelial plugs from WT and Δ*Chrpd3*, Δ*Chpho23*, Δ*Chsds3*, Δ*Chsin3* mutants were inoculated onto complete medium with xylose (CMX). After 4 days, Δ*Chrpd3*, Δ*Chpho23*, Δ*Chsds3*, Δ*Chsin3* mutants exhibited significantly reduced growth rates on CMX, with mycelial growth of Δ*Chrpd3* and Δ*Chsin3* mutants nearly abolished (Figure 2a,b). Additionally, as shown in Figure 2c, asexual reproduction was completely blocked in the Δ*Chrpd3*, Δ*Chpho23*, Δ*Chsds3*, Δ*Chsin3* mutants (Figure 2c). *C. heterostrophus* primarily disseminates in the field via asexual spores. Notably, compared with WT, the Δ*Chrpd3*, Δ*Chpho23*, Δ*Chsds3*, Δ*Chsin3* mutants caused significantly decreased virulence on detached maize leaves (Figure 2d).

### 2.3 *ChRPD3* , *ChPHO23* , *ChSDS3* and *ChSIN3* Are Necessary for the Response to Oxidative and Nitrosative Stress in *C. heterostrophus*

**FIGURE 3:**
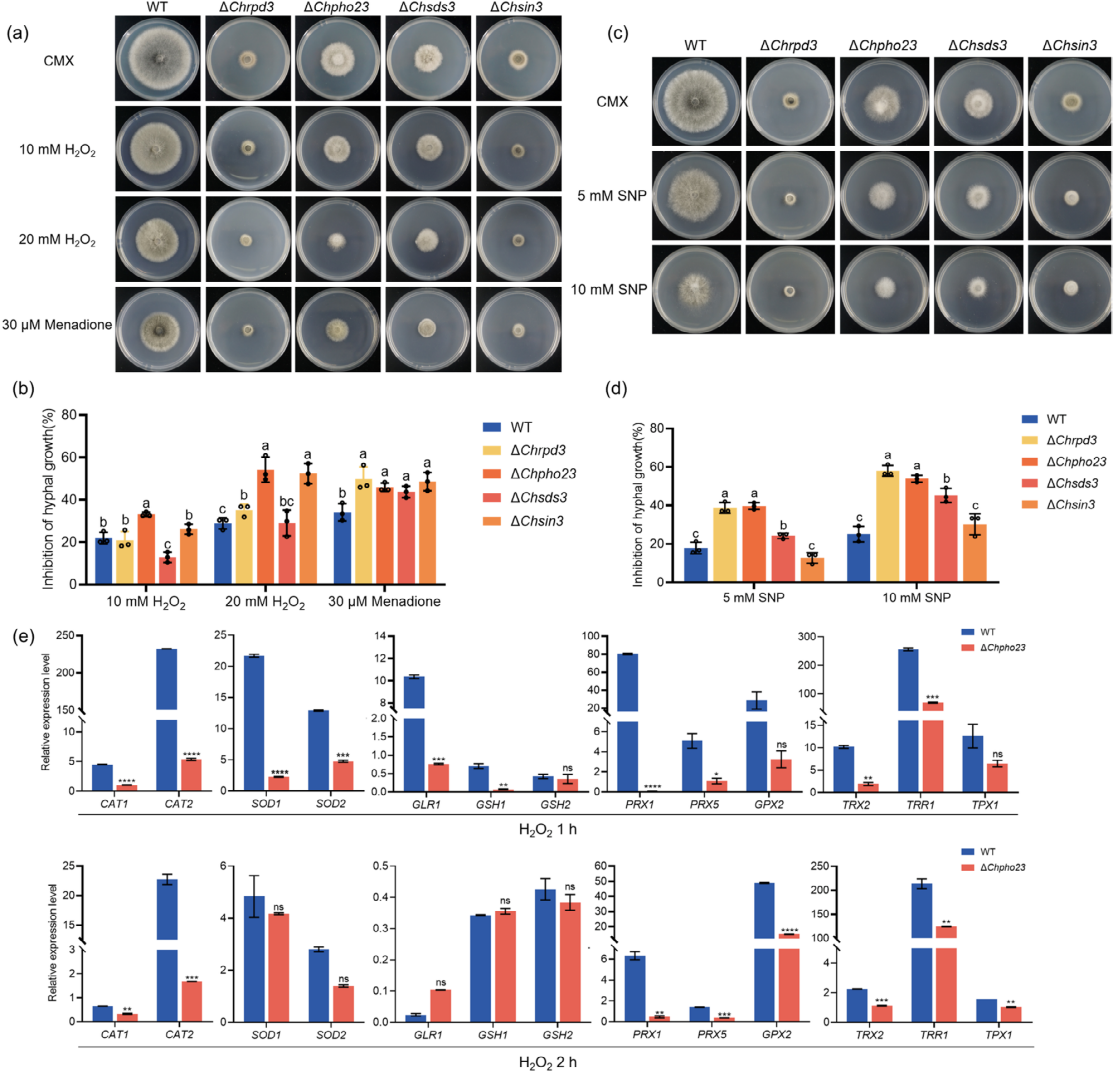
ChRPD3, ChPHO23, ChSDS3 and ChSIN3 were necessary for response to oxidative and nitrosative stress in *Cochliobolus heterostrophus*. (a) Sensitivity of indicated strains to H_2_O_2_ and menadione. (b) Statistical analysis of the inhibition rate of mycelial growth of each strain under oxidative stress. (c) Sensitivity of indicated strains to sodium nitroprusside (SNP). (d) Statistical analysis of the inhibition rate of mycelial growth of each strain under nitrosative stress. Data are displayed as mean values (±SE). Different letters above the columns indicate significant differences at *p* < 0.05, as determined by GraphPad Prism program *t* tests and multiple *t* tests, *n* = 3. The bars indicate the standard error of the mean. (e) Transcriptional levels of oxidative stress‐related genes in wild type (WT) and Δ*Chpho23* under H_2_O_2_ treatment. Each strain was cultured in complete medium with xylose (CMX) for 36 h and then transferred to CMX with 10 mM H_2_O_2_ for 1 h or 2 h. The *ChACTIN* gene was used as an internal control. Error bars indicate SEM from at least three independent experiments (marked with black dots on the bars). ** in (e) indicates significant differences based on *t* test (*p* < 0.01). *** in (e) indicates significant differences based on *t* test (*p* < 0.001). **** in (e) indicates significant differences based on *t* test (*p* < 0.0001).

To evaluate the roles of *ChRPD3*, *ChPHO23*, *ChSDS3* and *ChSIN3* in response to oxidative stress and NS in *C. heterostrophus*, we compared the mycelial radial growth of WT and Δ*Chrpd3*, Δ*Chpho23*, Δ*Chsds3* and Δ*Chsin3* mutants on CMX supplemented with H_2_O_2_, menadione, or sodium nitroprusside (SNP). Compared with WT, Δ*Chpho23* showed increased sensitivity to 10 and 20 mM H_2_O_2_, while Δ*Chsin3* and Δ*Chrpd3* were more sensitive to 20 mM H_2_O_2_ (Figure 3a,b). Additionally, all four mutants exhibited slower mycelial growth on CMX amended with 30 μM menadione (Figure 3a,b). Furthermore, 5 and 10 mM SNP significantly inhibited the mycelial growth of Δ*Chrpd3*, Δ*Chpho23* and Δ*Chsds3* compared to WT (Figure 3c,d). These results suggested that the Rpd3 complex is involved in responding to oxidative stress and NS in *C. heterostrophus*.

Given the important role of ChPho23 in responding to oxidative stress, the expression of 13 oxidative stress response genes was examined in WT and Δ*Chpho23* mutants upon H_2_O_2_ treatment. As shown in Figure 3e, except for *GSH2*, *GPX2* and *TPX1*, the expression of the remaining 10 genes (*CAT1*, *CAT2*, *SOD1*, *SOD2*, *GLR1*, *GSH1*, *PRX1*, *PRX5*, *TRX2* and *TRR1*) was dramatically reduced in Δ*Chpho23* mutants after 1 h of H_2_O_2_ treatment. After 2 h treatment, eight out of the 13 oxidative stress response genes (*CAT1*, *CAT2*, *PRX1*, *PRX5*, *GPX2*, *TRX2*, *TRR1* and *TPX1*) were significantly inhibited in Δ*Chpho23* mutants compared to WT.

### 2.4 ChPho23 and ChSds3 Interact Directly With ChHog1 to Mediate *C. heterostrophus* Response to Nitrosative Stress

**FIGURE 4:**
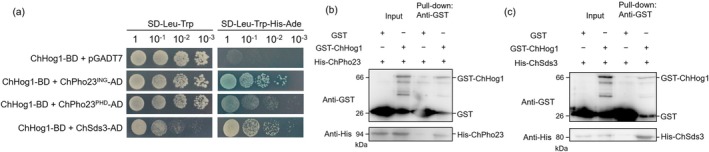
ChHog1 interacted with ChPho23 and ChSds3. (a) Yeast two‐hybrid validation of the interaction of ChHog1 with ChPho23 and ChSds3. (b) The interaction between ChHog1 and ChPho23 was verified by glutathione S‐transferase (GST) pull‐down assay. (c) The interaction between ChHog1 and ChSds3 was verified by GST pull‐down assay.

To further elucidate the regulatory mechanism of ChPho23 and ChSds3 in response to NS, we evaluated the connection between ChPho23/ChSds3 and the high osmolarity glycerol (HOG) pathway, which is known to adapt to a wide variety of environmental stimuli and responds to extracellular cues through sequential activation of a series of protein kinases. First, yeast two‐hybrid (Y2H) assays were used to identify the potential interactions between ChPho23/ChSds3 and ChHog1. As shown in Figure 4a, Y2H assays revealed that ChSds3 interacted with ChHog1. However, after more than five transformations, we were unable to successfully transform the pGADT7‐ChPho23 into yeast, suggesting that ChPho23 may be toxic to yeast cells. Therefore, the two functional domains of ChPho23 were cloned into pGADT7 to construct pGADT7‐ChPho23^ING^ and pGADT7‐ChPho23^PHD^ (Table S1). We found that both ChPho23^ING^ and ChPho23^PHD^ strongly interacted with ChHog1. The interaction was further validated by glutathione S‐transferase (GST) pull‐down assays, and the results were consistent with the Y2H data (Figure 4b,c). Taken together, these results imply that ChPho23 and ChSds3 may modulate the NS response by interacting with ChHog1 in *C. heterostrophus*.

### 2.5 ChHog1 Interacts With ChCrz1 to Modulate Nitrosative Response in *C. heterostrophus*

**FIGURE 5:**
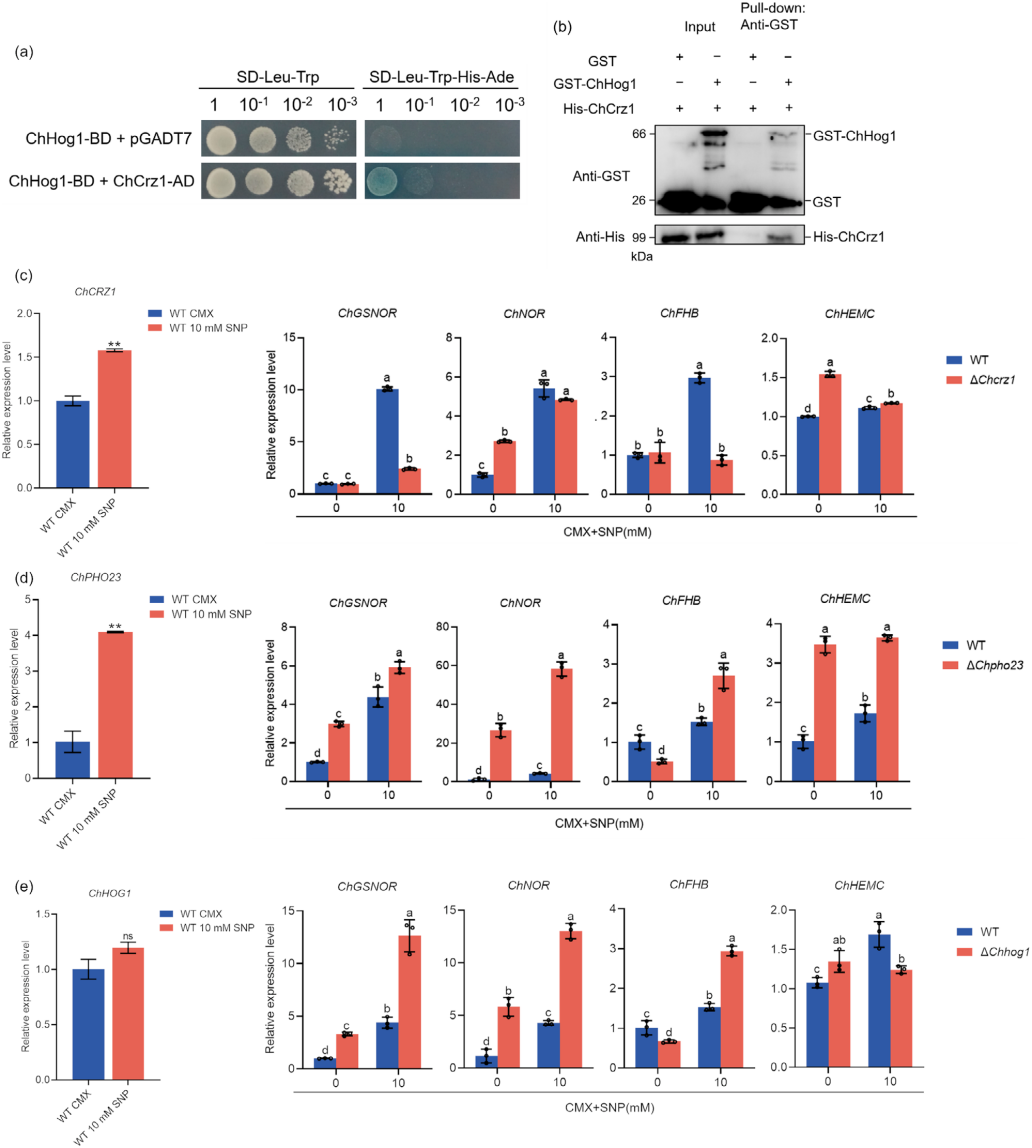
ChHog1 interacted with ChCrz1 to modulate the nitrosative stress response in *Cochliobolus heterostrophus*. (a) Yeast two‐hybrid assay validation of the interaction between ChHog1 and ChCrz1. (b) The interaction between ChHog1 and ChCrz1 was verified by glutathione S‐transferase (GST) pull‐down assay. (c) The expression of ChCRZ1 was induced by sodium nitroprusside (SNP) treatment, and ChCrz1 regulated the transcription of *ChGSNOR* and *ChFHB* in response to nitrosative stress in *C. heterostrophus*. (d) The expression of *ChPHO23* was induced by SNP treatment, and the expression of detoxification genes was changed in Δ*Chpho23* mutant compared to wild type (WT). (e) The expression of *ChHOG1* was not induced by SNP treatment, but the expression of detoxification genes was changed in Δ*Chhog1* mutant compared to WT. Each strain was cultured in complete medium with xylose (CMX) for 30 h and then transferred to CMX without or with 10 mM SNP for 2 h. The *ChACTIN* gene was used as an internal control. GraphPad Prism program's *t* tests and multiple *t* tests were used for the analysis of significant differences. The bars indicate SEM of three replications. ** in (c) indicates significant differences based on *t* test (*p* < 0.01).

To understand the molecular mechanism by which ChHog1 modulates the NS response in *C. heterostrophus*, we attempted to screen ChHog1‐interacting proteins in *C. heterostrophus* by using an affinity capture assay with ChHog1‐GFP as the bait. The WT strain C4 expressing GFP was used as a negative control. Among the candidate interacting proteins, we focused on ChCrz1, a C2H2‐type zinc‐finger transcription factor (COCC4DRAFT_144568). Further validation using Y2H and GST pull‐down assays confirmed that ChHog1 interacted with ChCrz1 (Figure 5a,b). In addition, the expression of *ChCRZ1* was induced by 10 mM SNP (Figure 5c).

Moreover, previous studies have identified five groups of NS response genes that are responsible for detoxifying NO in fungi. These include S‐nitrosoglutathione reductase (GSNOR), flavohaemoglobins (FHB), nitrosothionein, porphobilinogen deaminase (HEMC) and P450 nitric oxide reductases (NOR). BLASTp analysis revealed that *C. heterostrophus* possesses four putative NS response genes: *ChFHB* (XP_014081033.1), *ChGSNOR* (XP_014083174.1), *ChNOR* (XP_014077332.1) and *ChHEMC* (XP_014077178.1). The expression of *ChGSNOR*, *ChNOR* and *ChFHB* was induced by 10 mM SNP in the WT strain, but *ChGSNOR* and *ChFHB* were significantly inhibited in the Δ*Chcrz1* strain (Figure 5c). These results implied that ChCrz1 regulates the transcription of *ChGSNOR* and *ChFHB* in response to nitrosative stress in *C. heterostrophus*. Additionally, we also examined the expression of *ChFHB*, *ChGSNOR*, *ChNOR* and *ChHEMC* in Δ*Chpho23* and Δ*Chhog1* mutants. In contrast, the expression of *ChFHB*, *ChGSNOR* and *ChNOR* was significantly increased in Δ*Chpho23* and Δ*Chhog1* mutants (Figure 5d,e). This finding indicated that the deletion of *ChPHO23* or *ChHOG1* also affects the expression of detoxification genes in *C. heterostrophus*.

### 2.6 ChHog1 and ChCrz1 Are Necessary for the Nitrosative Stress Response in *C. heterostrophus*

**FIGURE 6:**
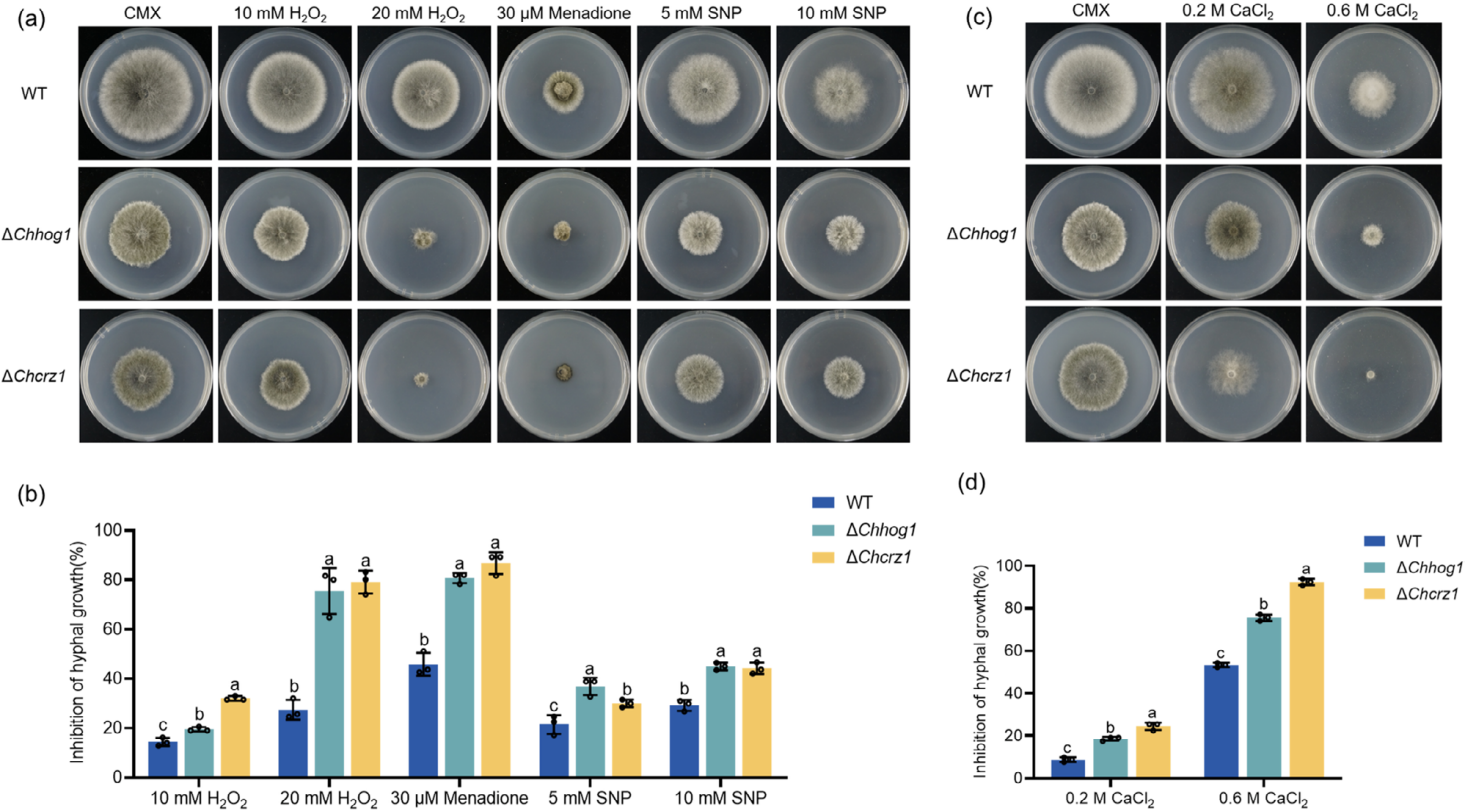
ChHog1 and ChCrz1 are necessary for stress responses. (a) The sensitivity of wild type (WT), Δ*Chhog1* and Δ*Chcrz1* were examined on complete medium with xylose (CMX) supplemented with H_2_O_2_, menadione or sodium nitroprusside (SNP). (b) The growth inhibition rate of WT, Δ*Chhog1* and Δ*Chcrz1* under various stress. (c) The growth of WT, Δ*Chhog1* and Δ*Chcrz1* was examined on the CMX supplemented with 0.2 M CaCl_2_ or 0.6 M CaCl_2_. (d) The growth inhibition rate of the WT, Δ*Chhog1* and Δ*Chcrz1* under osmotic stress. GraphPad Prism program's *t* tests and multiple *t* tests were used for the analysis of significant differences. The bars indicate SEM of three replications, different letters indicate significant differences.

In our previous studies, we characterised the roles of ChHog1 and ChCrz1 in virulence in *C. heterostrophus*. Both Δ*Chhog1* and Δ*Chcrz1* mutants exhibited significantly reduced virulence on detached maize leaves (Jia et al. 2023; Yu et al. 2024). To determine whether the decreased virulence of these mutants was related to sensitivity to SNP, the WT, Δ*Chhog1* and Δ*Chcrz1* mutants were inoculated on CMX amended with 5 or 10 mM SNP. As shown in Figure 6, SNP at both concentrations significantly inhibited the growth of the Δ*Chhog1* and Δ*Chcrz1* mutants compared to WT (Figure 6a,b). Additionally, Δ*Chhog1* and Δ*Chcrz1* mutants showed heightened sensitivity to H_2_O_2_, menadione and CaCl_2_ (Figure 6a–d). These observations suggest that deletion of *ChHOG1* and *ChCRZ1* increases the mutants' sensitivity to NS, oxidative stress and osmotic stress.

## 3 Discussion

Rpd3 encodes a catalytic component of class I HDACs, and Rpd3 complex consists of multiple subunits in fungi (Kurdistani and Grunstein 2003; Yang and Seto 2008; Zhang et al. 2020). Rpd3 involves removing acetylation from core histone H3 and H4 thus regulates gene expression and heterochromatin silencing in yeast (Rundlett et al. 1996). In pathogenic fungi, the components of the Rpd3 complex play crucial roles in growth, conidiation and virulence (Du et al. 2023; Lee et al. 2021; Lin et al. 2022; Ma et al. 2021; Wu et al. 2023; Xia, Wang, et al. 2024; Zhang et al. 2020). The Hog1‐MAPK cascade is highly conserved in filamentous fungi and acts as an intracellular signalling regulator to respond to external osmotic stress. The Hog1‐MAPK pathway has been found to regulate a diverse array of cellular responses, such as sexual and asexual development, cell growth, pathogenicity and secondary metabolite production (Frawley and Bayram 2020). The requirement of calcineurin for development and pathogenicity has been demonstrated across various pathogenic fungi (Liu et al. 2015; Park et al. 2019). As the major effector of calcineurin, the Crz1 (Calcineurin responsive zinc‐finger 1) deletion mutants exhibit increased sensitivity to osmotic stress and Ca^2+^, attenuated growth and decreased virulence (Cacciotti et al. 2024; Chen et al. 2019; Schumacher et al. 2008; Xia, Xia, et al. 2024; Zhang et al. 2009). In this study, the roles of *ChRPD3*, *ChPHO23*, *ChSDS3* and *ChSIN3* in growth, stress response and pathogenicity in *C. heterostrophus* were identified. In addition, ChPho23 and ChSds3 mediate *C. heterostrophus* response to NS via direct interaction with ChHog1. Moreover, ChHog1 interacts with ChCrz1 to modulate transcription of *ChGSNOR* and *ChFHB* to respond to NS in *C. heterostrophus*. To the best of our knowledge, this is the first study on the Rpd3 complex‐Hog1‐Crz1 in pathogenic fungi in response to NS.

As early as 1996, researchers found that Rpd3 has in vivo deacetylation activity at all sites examined in both core histones H3 and H4, thereby regulating gene transcription and silencing (Jia et al. 2023). To date, the functions of several Rpd3 complex components in growth, development and virulence have been reported in plant‐pathogenic fungi. For example, FgDep1, MoRpd3, FgSin3 and FgRpd3 all play important roles in the reproduction and infection process of pathogenic fungi (Lee et al. 2021; Lin et al. 2022; Xia, Wang, et al. 2024; Zhang et al. 2020). In addition, some components of the Rpd3 complex have been found to be involved in the regulation of *ATGs*. In yeast, transcriptional regulation of *ATG9* by the Pho23‐Rpd3 complex modulates the frequency of autophagosome formation (Jin and Klionshy, 2014). In *C. albicans* , the Rpd3 complex subunit Pho23 is also involved in the transcriptional regulation of autophagy (Du et al. 2023). In this study, we found that the expression of autophagy‐related genes changed after the knockout of Pho23 and Rpd3, but the changes in different autophagy‐related genes were inconsistent, with some showing up‐regulation and others showing down‐regulation. However, our study is the first report on the effect of the Rpd3 complex on the response of pathogenic fungi to NS. Although there have been previous reports on the regulation of the Rpd3 complex in response to osmotic stress, exposure of yeast cells to high osmolarity results in rapid activation of the MAPK Hog1; then, Hog1 interacts physically with Rpd3 in vivo and in vitro, targeting the deacetylase to specific osmostress‐responsive genes (De Nadal et al. 2004). It is well known that Hog1 is crucial for pathogenic fungi to resist osmotic stress, but research on Hog1's role in resisting NS is still limited. In this study, we clarified that the Rpd3 complex can interact with Hog1, thereby influencing the response of *C. heterostrophus* to NS. In future, we will further investigate whether the Rpd3 complex can deacetylate Hog1.

The transcription factor Calcineurin‐Responsive Zinc finger 1 (CRZ1) can modulate several pathways and orchestrate cellular responses to different types of environmental insults such as osmotic stress, oxidative stress and membrane disruptors (Cacciotti et al. 2024). As previously elucidated, the CRZ1 maintains sway over fungal growth, development and pathogenicity. The absence of CRZ1 causes compromised mycelial growth and virulence in *Valsa pyri*, *M. oryzae* , *B. cinerea* and *F. graminearum* (Chen et al. 2019; He et al. 2016; Schumacher et al. 2008; Zhang et al. 2009). In addition, CRZ1 governs a range of target genes that are responsible for sustaining cell wall integrity, maintaining ion equilibrium and managing protein degradation. In this study, the expression of *ChCRZ1* was induced by SNP and the deletion of *ChCRZ1* significantly inhibited the expression of SNP detoxification genes. However, the deletion of *ChPHO23* or *ChHOG1* caused increased expression of SNP detoxification genes. We speculate that, in addition to the ChCrz1 pathway, ChPho23 and ChHog1 may also regulate the expression of SNP detoxification genes through other pathways. In *F. graminearum*, RNA‐seq data revealed that FgCrz1 regulates the expression of a set of non‐*CYP51* genes that are associated with tebuconazole sensitivity. Moreover, the HOG and calcium‐calcineurin pathway act coordinately to orchestrate tebuconazole sensitivity and pathogenicity in *F. graminearum* (Wang et al. 2023). Furthermore, FvHog1 can directly phosphorylate FvCrz1 to regulate Ca^2+^ homeostasis in *F. verticillioides*. However, FvHog1 has no direct interaction with FvCrz1. What differs from the previous study is that ChHog1 can directly interact with ChCrz1 in *C. heterostrophus*. Additionally, although FvHog1 has no direct interaction with FvCrz1, the phosphorylated FvHog1 enters the nucleus to regulate the expression of FB1 biosynthesis *FUM* genes. In this study, we found that both ChPho23 and ChSds3 interacted with ChHog1, and ChHog1 interacted with ChCrz1. We speculate that both ChPho23 and ChSds3 may act as the stress receptors, then pass the signal to ChHog1 and that ChHog1 enters the nucleus to regulate the expression of detoxification genes through interaction with ChCrz1. In future, we will test if ChPho23 and ChSds3 can directly deacetylate ChHog1.

This study further enriches our understanding of Crz1 and provides important evidence for the future development of novel fungicides targeting Crz1. In the future, we will verify whether ChHog1 has a direct phosphorylation effect on ChCrz1.

## 4 Experimental Procedures

### 4.1 Fungal Strains and Growth Condition

The wild‐type strain *C. heterostrophus* C4 (*Tox1* ^+^, *MAT1‐2*, ATCC 48331) was used as the parental strain. Unless otherwise specified, all strains were grown on complete medium with xylose (CMX) at 25°C in the dark. For NS testing, mycelial plugs were inoculated onto CMX supplemented with SNP (Aladdin).

### 4.2 Construction of Mutants and PCR Verification of Gene Deletion

The generation of deletion mutants and complemented strains was carried out as previously described (Yu et al. 2024). The polyethylene glycol (PEG)‐mediated protoplast transformation method was used for gene deletion in *C. heterostrophus*. Briefly, the 5′ and 3′ flanking regions were amplified using primer pairs F1/R1 and F2/R2, respectively, and fused with the *HPH* fragment containing the *TrpC* promoter and terminator regions, which were amplified from the plasmid pUCATPH using primers M13F and M13R. The *C. heterostrophus* transformation was conducted as described in Yu et al. (2022); hygromycin was used as the selection marker, and mutant candidates were verified by diagnostic PCR.

For complementation, the WT genes with 5′ and 3′ flanking regions, a fragment further 3′ of the target gene 3′ flanking sequence, and the *NPTII* gene from plasmid pII99 were amplified and co‐transformed into the mutants. The transformants were screened with geneticin and verified with diagnostic PCR.

### 4.3 Phenotype Assay

For asexual conidia production, 7‐day‐old cultures of each strain grown on CMX were rubbed completely to harvest conidia, which were then filtered through four layers of cheesecloth to remove mycelial debris. Conidia were counted using a haemocytometer, and the conidial suspensions were adjusted to 5 × 10^4^ conidia/mL. To compare the mycelial growth of mutants to WT, 5‐mm mycelial plugs were inoculated onto CMX and incubated at 25°C in the dark. The colony diameters were measured on Days 1–4. For stress adaptation assays, WT and mutant strains were inoculated onto CMX or minimal medium (MM) supplemented with H_2_O_2_, menadione or SNP.

### 4.4 Virulence Test

To test the virulence of WT and mutant strains, 5‐mm mycelial plugs of each strain were inoculated on 3‐week‐old maize ( *Zea mays* ‘B73’) detached leaves. The inoculated maize leaves were placed in a mist chamber at 25°C with 85% humidity for 24 h, then transferred to 25°C under a 16 h light/8 h dark cycle. The lesion size was evaluated with ImageJ on the third day, and each experiment was repeated three times.

### 4.5 Y2H Assays

To verify the protein–protein interactions, a Y2H assay was performed. The coding sequences of ChSds3, ChUme6 and ChPho23 were amplified from cDNA of C4 using the corresponding primers and cloned into pGADT7 to construct the prey plasmid. The coding sequences of ChSin3, ChSds3, ChUme6, ChHog1 and ChCrz1 were also amplified from cDNA of C4 and cloned into pGBKT7 to construct the bait plasmids. Both bait and prey vectors were verified by sequencing and co‐transformed into the yeast strain AH109 following the lithium acetate method. After growing on synthetic dropout (SD) medium lacking Leu and Trp, the yeast cells were serially diluted and transferred onto SD without Leu, Trp, His and Ade to confirm the protein–protein interactions. The interaction between pGADT7‐T and pGBKT7‐53 was used as a positive control, and the interaction between pGADT7‐T and pGBKT7‐Lam was used as a negative control.

### 4.6 GST Pull‐Down Assay

To perform GST pull‐down assays, the full‐length coding sequences of ChSds3, ChPho23 and ChCrz1 were amplified from the cDNA of C4 and cloned into pET‐32a to generate His‐tagged plasmids. In addition, the full‐length coding sequence of ChHog1 was amplified and cloned into pGEX to generate GST‐tagged plasmids. Both His‐tagged and GST‐tagged plasmids were expressed in *Escherichia coli* BL21 and purified. To determine the interaction between His‐tagged proteins and GST‐tagged proteins, GST‐tagged proteins were incubated with GST Sepharose beads for 4 h at 4°C, followed by the addition of His‐tagged proteins for another 4 h at 4°C. The beads were washed five times with Tris‐buffered saline (TBS), and the proteins were eluted. GST was used as a negative control. Finally, the protein interactions were determined by anti‐His and anti‐GST antibodies.

### 4.7 RNA Extraction and Reverse Transcription‐Quantitative Real‐Time PCR Analysis

Total RNA was extracted from hyphae harvested from WT and mutant strains grown on CMX plates for 7 days, and each sample was ground into fine powder in liquid nitrogen and extracted with TRIzol reagent (Invitrogen). Genomic DNA was removed using the Ambion TURBO DNA‐free kit (Applied Bio Systems). Reverse transcription‐quantitative real‐time PCR was conducted to measure the expression levels of target genes with a PrimePro 48 real‐time detection system (Techne Inc.). Each experiment included three biological replicates.

### 4.8 Screening of Interaction Proteins of ChHog1

The WT::Hog1‐GFP strain was ground into powder in liquid nitrogen, and cell lysis buffer and protease inhibitor were added, then centrifuged at 15,000 *g* for 10 min at 4°C. The supernatant was mixed with GFP beads and incubated at 4°C for 2 h. After incubation, the mixture was centrifuged at 2500 *g* for 2 min at 4°C, and the supernatant was discarded. The samples were then used for protein preparation, followed by SDS‐PAGE detection. The gels were sent to Cosmos Wisdom (Hangzhou, China) for spectrometry analysis.

### 4.9 Statistical Analysis

For statistical analysis, GraphPad Prism program's *t* tests and multiple *t* tests were used for the analysis of significant differences. All data shown are the mean ± SEM.

## Author Contributions

**Jinyu Fan:** investigation. **Jun Hu:** investigation. **Dan Li:** investigation. **Yuanyuan Tian:** investigation. **Mengjiao Jia:** investigation. **Tianye Liang:** writing – original draft. **Hongyu Pan:** conceptualization. **Xianghui Zhang:** conceptualization, supervision, writing – original draft.

## Conflicts of Interest

The authors declare no conflicts of interest.

## Supporting information


Table S1.

## Data Availability

The data that support the findings of this study are available from the corresponding author upon reasonable request.
