# Supplementary material for: Two Subunits of the Rpd3 Histone Deacetylase Complex of Cochliobolus heterostrophus Are Essential for Nitrosative Stress Response and Virulence, and Interact With Stress‐Response Regulators ChHog1 and ChCrz1
Source: Mol Plant Pathol. 2025 Aug 7;26(8):e70131. doi: 10.1111/mpp.70131 (PMC12330922; doi:10.1111/mpp.70131)
Supplement: Supplementary file 1 — Table S1. [file MPP-26-e70131-s001.docx]

Table S1 The sequence of Pho23ING and Pho23PHD used in Y2H

| Domain | Sequence |
| --- | --- |
| Pho23ING | GCCCTCACACACTTCACAGGCGCCGTCGATGCGTTCCCCAAGGAAATTATCAAGCACTTTTCCATGTTCAAGGAGGTCGAGGCTAAACTACACGACCCGGAACACTTGCTCGAGGAGTTGCTCGACGAGATTGCCCACCAGCCCGTCACAACAAGGGCCCAAGCATCGGCCGCCGGCCAGAATGCAGCGGGAGCGGAAAACAGCGCCAACCCGTCCCAGAACCCTGAAACCCTCTCCCCGGAAGAGCAGGCCACAATACGAAAGCGCCAGCTCTTCTATCGCCTGCGCATGCTCATCGCAAACATGCTCCCTACGCTCGACGAGAAACTGGTAGTGCTTCAGGGCGCAAAAGCCACCAAAGACAAAGGCCTCATGCGGATGCACCACTCATACGCGCAGCTC |
| Pho23PHD | TACTGCTATTGCAATGAAGTGTCATATGGCAACATGATTGCCTGTGACAACGATGACTGCCCGCGCGAGTGGTTCCATCTCGGCTGCGTACACCTGGAGAAGCCGCCTACGGGGCGGGCGAAGTGGTTCTGCAGCGACGAGTGC |
